# Prevalence and Antibiogram Pattern of *Klebsiella pneumoniae* in a Tertiary Care Hospital in Makkah, Saudi Arabia: An 11-Year Experience

**DOI:** 10.3390/antibiotics12010164

**Published:** 2023-01-12

**Authors:** Naif A. Jalal, Abdulrahman M. Al-Ghamdi, Aiman M. Momenah, Sami S. Ashgar, Farkad Bantun, Fayez Saeed Bahwerth, Sumyya H. Hariri, Ayman K. Johargy, Abeer A. Barhameen, Hamdi M. Al-Said, Hani Faidah

**Affiliations:** 1 Department of Microbiology, Faculty of Medicine, Umm Al-Qura University, Makkah 21955, Saudi Arabia; 2 Department of Molecular Biology, King Faisal Hospital, Makkah 21955, Saudi Arabia

**Keywords:** *Klebsiella pneumoniae*, antibiotic resistance, MDR, ESBL, Saudi Arabia, Makkah, antibiogram pattern

## Abstract

Infectious disease is one of the greatest causes of morbidity and mortality worldwide, and with the emergence of antimicrobial resistance, the situation is worsening. In order to prevent this crisis, antimicrobial resistance needs to be monitored carefully to control the spread of multidrug-resistant bacteria. Therefore, in this study, we aimed to determine the prevalence of infection caused by *Klebsiella pneumoniae* and investigate the antimicrobial profile pattern of *K. pneumoniae* in the last eleven years. This retrospective study was conducted in a tertiary hospital in Makkah, Saudi Arabia. Data were collected from January 2011 to December 2021. From 2011 to 2021, a total of 61,027 bacterial isolates were collected from clinical samples, among which 14.7% (n = 9014) were *K. pneumoniae*. The antibiotic susceptibility pattern of *K. pneumoniae* revealed a significant increase in the resistance rate in most tested antibiotics during the study period. A marked jump in the resistance rate was seen in amoxicillin/clavulanate and piperacillin/tazobactam, from 33.6% and 13.6% in 2011 to 71.4% and 84.9% in 2021, respectively. Ceftazidime, cefotaxime, and cefepime resistance rates increased from 29.9%, 26.2%, and 53.9%, respectively, in 2011 to become 84.9%, 85.1%, and 85.8% in 2021. Moreover, a significant increase in the resistance rate was seen in both imipenem and amikacin, with an average resistance rate rise from 6.6% for imipenem and 11.9% for amikacin in 2011 to 59.9% and 62.2% in 2021, respectively. The present study showed that the prevalence and drug resistance of *K. pneumoniae* increased over the study period. Thus, preventing hospital-acquired infection and the reasonable use of antibiotics must be implemented to control and reduce antimicrobial resistance.

## 1. Introduction

*Klebsiella pneumoniae* (*K. pneumoniae*) is a Gram-negative encapsulated bacterium that colonizes multiple sites of the human body, including the gastrointestinal tract, respiratory tract, oral cavities, and skin [1]. *K. pneumoniae* is an opportunistic pathogen and a major cause of hospital-acquired infections, including bloodstream infections, urinary tract infections, and pneumonia [2]. To survive the lethal effects of antibiotics, *K. pneumoniae* developed multiple resistance mechanisms, such as modifying the target site, drug inactivation, reduced cell permeability, and efflux pump activation [3,4]. For example, some strains of *K. pneumoniae* can survive and overcome the effect of β-lactam antibiotics by producing extended-spectrum beta-lactamase (ESBL) enzymes. ESBLs can hydrolyze and inactivate β-lactam antibiotics, including penicillins, cephalosporins (first-, second-, and third-generation), and aztreonam, but not cephamycins or carbapenems [4]. However, the effect of ESBL enzymes can be overcome by β-lactams inhibitors, such as clavulanic acid [3]. ESBLs are encoded by transferable plasmid-mediated genes, such as TEM, SHV, and CTX-M [5]. Since the identification of the first ESBL-producing bacteria in 1980, the incidence of ESBL-producing organisms has increased drastically to become a significant public health threat [6,7]. Recently, it has been noticed that ESBL-producing bacteria exhibit a high resistance rate to other antibiotic classes, such as fluoroquinolones, sulfonamides, and aminoglycosides [8]. In addition to ESBL, *K. pneumoniae* acquired resistance to carbapenems through three major mechanisms: enzyme production, efflux pumps, and porin mutations [9]. Among these, enzyme production (carbapenemases) is the most dominant resistance mechanism [9]. Furthermore, colistin resistance was also observed in *K. pneumoniae* and other Gram-negative bacteria. The mechanism of colistin resistance is generally through the modification of the lipopolysaccharide (LPS) outer membrane structure, which would lower the binding affinity of colistin to the LPS [10]. Due to this increase in antibiotic resistance, multiple studies were conducted worldwide to monitor the antibiotic resistance of *K. pneumoniae* [11].

In Saudi Arabia, the surveillance of antibiotic resistance of *K. pneumoniae* revealed an increase in the resistance rate among different antibiotic classes. For example, in Aseer (south), several studies conducted between 2015 and 2019 showed an increase in the resistance rate against penicillin and cephalosporin. Nearly all the isolates were resistant to ceftazidime, piperacillin, and ampicillin [12,13]. Similarly, a previous study in Riyadh (center) showed that 55% of *K. pneumoniae* were capable of producing ESBL and exhibited a high resistance rate to ceftazidime (95%) and cefotaxime (97%) [5]. In addition, during a four-year surveillance of *K. pneumoniae* in Al-Medina (north), Saudi Arabia, over half of the isolates were found to be resistant to different classes of antibiotics (with an average of 61.7%). Among carbapenems, 38.7% and 46.1% resistance rates were found towards imipenem and meropenem, respectively [14]. Moreover, the study revealed a high resistance to colistin and tigecycline. Although the result of colistin resistance was not reliable due to the low sample size, this finding is worrying because of the limited options for treating infection caused by multidrug-resistant *K. pneumoniae* [14,15]. The aforementioned studies indicated a continuous increase in the antibiotic resistance rate of *K. pneumoniae*, which requires urgent and continuous monitoring. Therefore, this study aimed to determine the prevalence of *K. pneumoniae* infections and the resistance trend over eleven years in Makkah, Saudi Arabia.

## 2. Results

### 2.1. Patient and Samples Demographics

This retrospective study was carried out to determine the burden of infections caused by *K. pneumoniae* over eleven years, from January 2011 to December 2021. Out of 61,027 isolates found during the study period, 9014 (14.7%) were *K. pneumoniae*. The results showed an increase in *K. pneumoniae* isolates over the years, with an annual number of isolates ranging from 622 to 1131 and an average of 819.5 ± 205.7. The detection rates of *K. pneumoniae* increased from 7.7% in 2011 to 25.9% in 2020. However, the results revealed that the rates of *K. pneumoniae* isolates drastically reduced from 25.9% in 2020 to 17.9% in 2022, which could be due to the strong implementation of infection control policies following the COVID-19 pandemic (Table 1).

**Figure 1:**
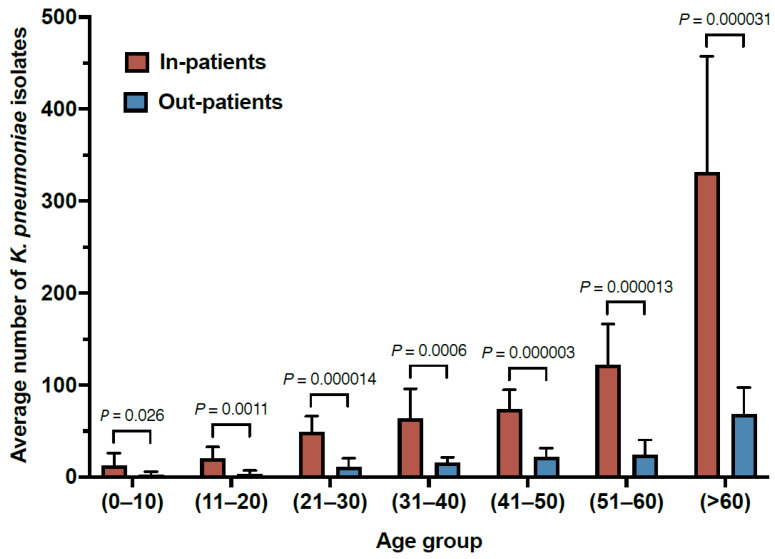
The distribution of *K. pneumoniae* isolates from inpatient and outpatient departments from 2011–2021.

Throughout the study period, the results showed a significantly higher rate of *K. pneumoniae* isolated from inpatients (mean: 672.5 ± 193.6) compared to outpatients (mean: 147 ± 51.9) (Table 1 and Figure 1). In addition, higher rates of *K. pneumoniae* isolates were obtained from male subjects compared to females (*p* value ≤ 0.0001) (Table 1). The results also showed a direct correlation between age and the rates of *K. pneumoniae* isolates (Figure 1). Collectively, these results may suggest that prolonged hospitalization and weakened immunity due to aging are potential risk factors for *K. pneumoniae* infection.

**Figure 2:**
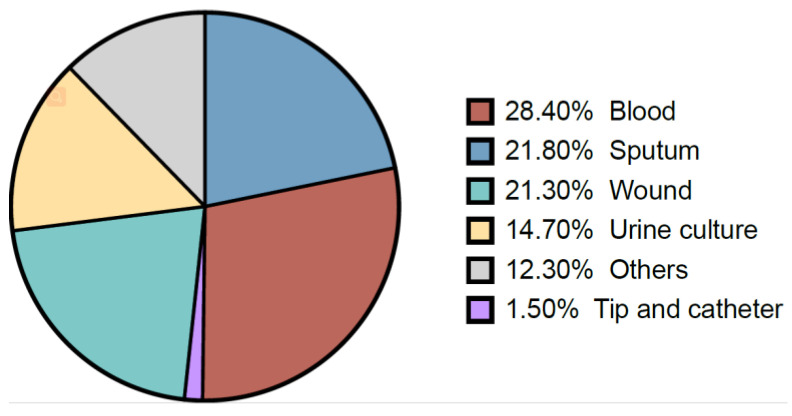
The proportion of different specimen types of *K. pneumoniae*.

**Figure 3:**
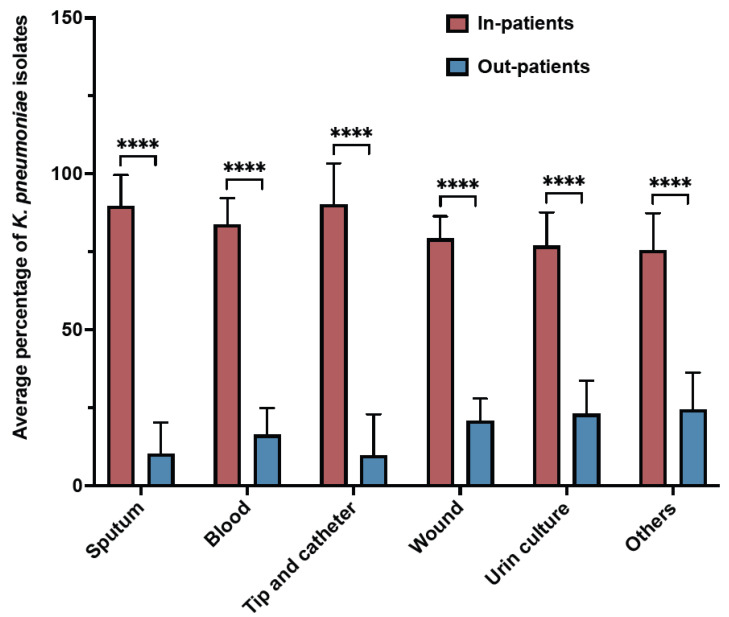
Samples distribution of *K. pneumoniae* isolates from inpatients and outpatients over 11 years. **** *p* ≤ 0.000001.

During the study period, *K. pneumoniae* isolates were reported mostly from blood samples (28.4%), followed by sputum (21.8%), wounds (21.3%), urine (14.7), and tip and catheter (1.5%) (Figure 2). Overall, the results showed a gradual increase in the percentage of detection of *K. pneumoniae* isolates from different samples over the years (Table 2). Regardless of the sample origin, *K. pneumoniae* isolates were significantly higher from inpatient specimens (mean: 82.55 ± 6.3) compared to outpatients (mean: 17.45 ± 6.4) (Figure 3). For example, 83.65% of *K. pneumoniae* isolated from blood samples were obtained from inpatient specimens, and the remaining 16.35% were from outpatients (Figure 3).

### 2.2. Antimicrobial Resistance Profile of K. pneumoniae

**Figure 4:**
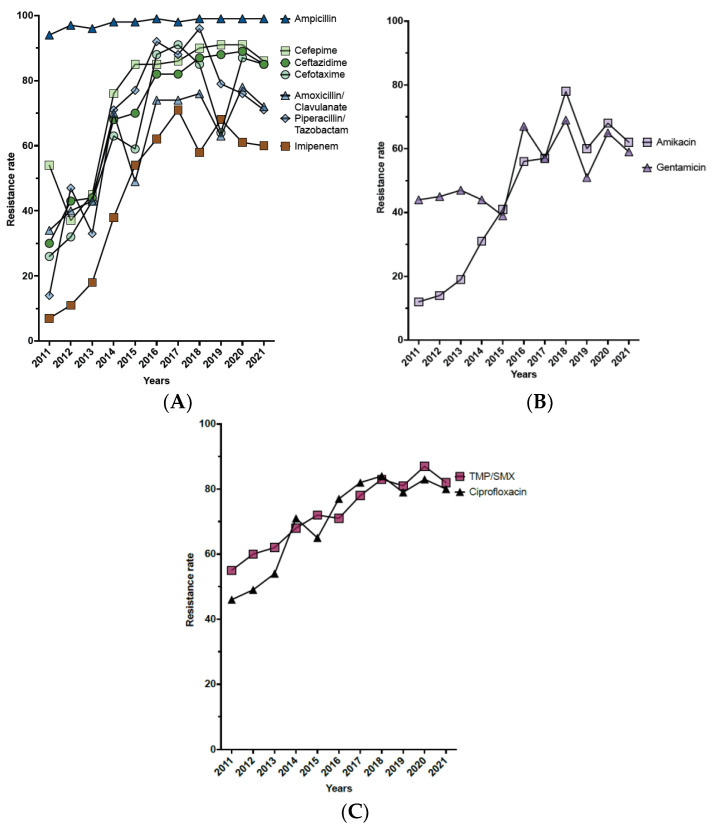
Schematic demonstration of the antimicrobial resistance trends of *K. pneumoniae*. (**A**). Evolution of resistance to β-lactam antibiotics. (**B**). Evolution of resistance to aminoglycoside. (**C**). Evolution of resistance to ciprofloxacin and cotrimoxazole (trimethoprim-sulfamethoxazole).

The analysis of the antimicrobial susceptibility pattern of *K. pneumoniae* revealed a sharp increase in the resistance rate in most of the tested antibiotics (Table 3, Figure 4A–C). Throughout the duration of the study, the highest resistance rate was observed in ampicillin (97.6%), and the lowest resistance rate was for tigecycline (15.7%) (Table 3). The complex of β-lactam/β-lactamase-inhibitor antibiotics (amoxicillin/clavulanate and piperacillin/tazobactam) showed a significant gradual increase in the resistance rate over the eleven years (Table 3 and Figure 4A). Similarly, a sharp increase in resistance was observed in the third- and fourth-generation cephalosporins (ceftazidime, cefotaxime, and cefepime) (Table 3 and Figure 4A). Among the cephalosporin antibiotics, cefotaxime showed the lowest resistance rate (68.6%), followed by ceftazidime (74.9%), and cefepime (81.0%) (Table 3). Notably, imipenem was the most sensitive antibiotic among the β-lactam antibiotic family in the first two years of the study; however, the resistance rate of imipenem escalated progressively to reach 60% in 2021 (Table 3 and Figure 4A). Like β-lactam antibiotics, aminoglycosides (amikacin and gentamicin) showed a significant increase in the resistance rate over the study period. Over the eleven years, the average resistance rate of amikacin was 47.8%, whereas that of gentamicin was 54.9% (Table 3 and Figure 4B). Similarly, ciprofloxacin and cotrimoxazole (trimethoprim-sulfamethoxazole) showed a significant increase in the resistance rate over time, with average resistance rates of 71.6% and 74.9%, respectively (Table 3 and Figure 4B). In addition, the results showed that the overall resistance rates of colistin and tigecycline for the study period were 25.4% and 15.7%, respectively. However, the results for both antibiotics were not statistically significant due to the low sample size (Table 3).

**Figure 5:**
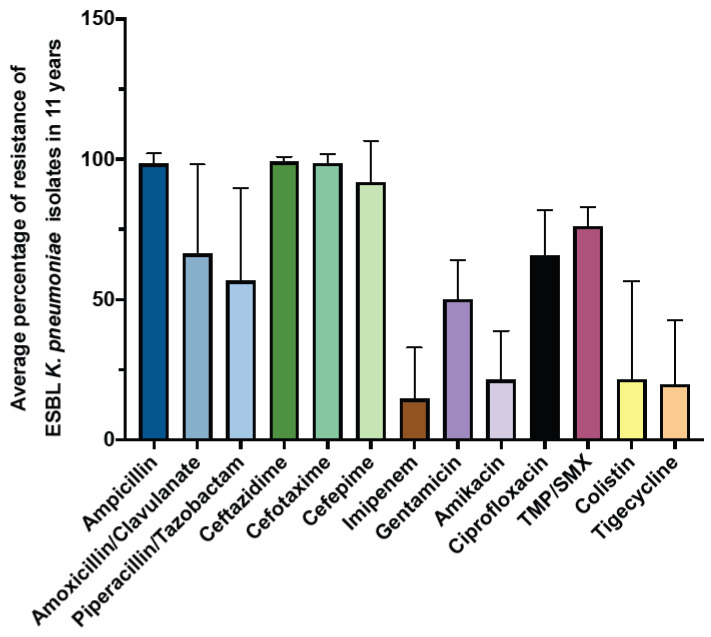
The average percentage of resistance of ESBL *K. pneumoniae* over 11 years.

Next, we examined the prevalence and antimicrobial susceptibility pattern of the extended-spectrum β-lactamase (ESBL) *K. pneumoniae* (Figure 5). The results showed that the average prevalence of ESBL throughout the study period was 21.3%. In addition, our result showed that ampicillin and cephalosporin exhibited low activity against ESBL *K. pneumoniae*, with an average resistance rate of more than 90%. In contrast, the most effective antibiotics against ESBL *K. pneumoniae* were imipenem, with an average resistance rate of 14.63 ± 18.20, followed by tigecycline (19.67 ± 22.97), amikacin (21.31 ± 17.44), and colistin (21.52 ± 35.01) (Figure 5).

## 3. Discussion

Antimicrobial resistance (AMR) is a global threat to human health. More than 1 million deaths could have been avoided in 2019 if sensitive drugs had been replaced by resistant drugs in the world [16]. The misuse and overuse of antibiotics, together with the ability of resistant microbes to transmit from person to person, have significantly amplified the problem of AMR [17,18]. For instance, the emergence of multidrug-resistant *K. pneumoniae* was directly linked to the misuse and overuse of antibiotics when treating hospitalized patients [19]. Therefore, active surveillance of AMR trends is essential worldwide. In Saudi Arabia, only a limited number of studies were conducted to monitor the AMR trends of *K. pneumoniae*. Thus, in this study, we helped to fill the gap by investigating the prevalence and trends of AMR of *K. pneumoniae*.

Our results showed a gradual increase in the rate of infections with *K. pneumoniae* over the years. The prevalence of *K. pneumoniae* dramatically increased from 7.7% in 2011 to a peak in 2020 of 25.9%. The average rate of infections with *K. pneumoniae* in this study was 14.7% (n = 9014) over 11 years. This rate of infections with *K. pneumoniae* is lower than that reported previously in other regions in Saudi Arabia. For example, the prevalence of infections with *K. pneumoniae* was 39% and 18.6% in Asser and Bisha, respectively [13,20]. Nevertheless, the prevalence of infections with *K. pneumoniae* in Saudi Arabia is similar to that reported worldwide (i.e., 18.8 to 87.7% in Asia and 5 to 35% in Western countries) [21]. In this study, the majority of *K. pneumoniae* isolates were found to be from male patients, with an average of 58.5% ± 4.9. However, it is crucial to notice that the majority of total isolates were also obtained from male patients with an average of 59.3 ± 2.8. This result is in agreement with the previous studies, where the majority of *K. pneumoniae* isolates were obtained from male patients [20,22].

In addition, a direct correlation between the rate of infections with *K. pneumoniae* and age was observed. A similar observation was reported in Aseer, Saudi Arabia, where 42% of *K. pneumoniae* infections were in patients >60 years old [13]. This is possibly due to the decline of the immune system with aging, which allows the bacteria to multiply and cause infection [23]. Moreover, this study showed a significantly higher rate of *K. pneumoniae* isolated from inpatients compared to outpatients. Taken together, these findings may suggest that weakened immunity due to aging and extended hospitalization are potential risk factors for *K. pneumoniae* infection.

With regard to *K. pneumoniae* detection from different samples, the results showed that the highest rates were in blood samples, followed by sputum. This finding is consistent with the previous reports from Saudi Arabia, in which the majority of *K. pneumoniae* were isolated from blood and respiratory specimens [12]. The present finding relies on the fact that *K. pneumoniae* is one of the main causative agents of bloodstream infection and respiratory tract infection, and it should be taken into consideration during the diagnosis of respiratory infection [24,25]. 

In agreement with national and international reports, our results revealed the emergence of antimicrobial resistance, where *K. pneumoniae* is resistant to the most commonly used antibiotics [11,20,26]. Throughout the study period, ampicillin showed the highest resistance rate among the tested antibiotics, with a 97.6% average resistance rate. Similar results were reported in previous studies in Saudi Arabia; for example, in Aseer and Medina, ampicillin showed a 100% and 99.9% resistance rate, respectively [13,14]. Similarly, the results revealed that the resistance rate of *K. pneumoniae* to third- and fourth-generation cephalosporin rose significantly with 74.9% (n = 4451), 68.6% (n = 2244), and 81.0% (n = 4451) for ceftazidime, cefotaxime, and cefepime, respectively. This finding is in agreement with previous reports from Saudi Arabia, which indicated an increase in the resistance rate of *K. pneumoniae* to cephalosporin antibiotics [13,14]. The same finding was also reported in China and other countries worldwide, which suggests a global rise in resistance to third- and fourth-generation cephalosporins by *K. pneumoniae* [11,27]. A similar increase in the resistance rate of *K. pneumoniae* was also detected for amikacin. Interestingly, during the earlier years of the study, amikacin was one of the most effective antibiotics, with a resistance rate of 11.9%. Unfortunately, the rate of resistance started to increase aggressively over the period of study to reach its peak of 77.7% in 2018. A similar finding was reported in Medina, Saudi Arabia, where the resistance rate of *K. pneumoniae* to amikacin increased from 28.9% to 39.7% over the four-year duration of the study [14].

Carbapenem is a potent antibiotic to treat infections caused by Gram-negative bacteria. It has been used for a long time as a first choice to treat infections caused by ESBL-producing bacteria. However, resistance to carbapenems is alarmingly increasing, resulting in a future global crisis [28,29]. In this study, we assessed the resistance profile to imipenem, which showed a remarkable increase in the resistance rate over the study period from only 6.6% in 2011 to 59.9% in 2021. The rapid increase in carbapenem-resistant Enterobacteriaceae (CRE) has been reported both locally and globally [12,13,14,30,31]. This increase in CRE is linked to the increased use and consumption of β-lactamase inhibitors [32,33]. 

Colistin was an antibiotic commonly used to treat infections caused by Gram-negative bacteria in the 1960s and 1970s [34]. However, as a result of colistin renal- and neurotoxicity, the drug was discontinued [35]. Nevertheless, due to the emergence of CRE, the use of colistin has become a necessity since colistin is the last resort to treat CRE infections [15]. Consequently, even a low resistant rate could result in a threatening situation in the future. In this study, no significant change in the resistance rate of colistin was observed over the years, which could be due to the low number of tested isolates. Nevertheless, the resistance pattern showed some fluctuation during the study period. Multiple studies revealed a notable rise in colistin resistance both in the Kingdom of Saudi Arabia and globally. For example, the prevalence of colistin resistance in Medina, Ha’il, and Asser (Saudi Arabia) was reported to be 40.7%, 26.80%, and 35.10%, respectively [13,14,36]. Internationally, the prevalence of colistin resistance varies widely, with 13% in the USA and 73% in Italy [37,38]. 

In conclusion, our study revealed that the prevalence of *K. pneumoniae* increased significantly during the study period. In addition, we observed a significant increase in the resistance rate to nearly all of the tested antibiotics (except colistin and tigecycline) over the study years. Unfortunately, no significant decline in the resistance pattern was found in any of the tested antibiotics. Therefore, the reasonable use of antibiotics, an active antimicrobial stewardship program, and intensive educational programs for physicians as well as clinical pharmacists must be implemented to control and reduce antimicrobial resistance among *K. pneumoniae* and other multidrug-resistant bacteria. This study was limited to a single center from Makkah in Saudi Arabia. This investigation was conducted with a total sample size of 61,027 isolates collected over 11 years. Thus, we can comfortably state that this is a representative sample of Makkah. However, this does not exclude the need to perform similar studies in other regions and include more cities and centers to obtain a representative sample of the country. Nevertheless, such a study will need more funding and time to perform. Due to the unavailability of antimicrobial prescription patterns over the study period, we were unable to analyze the impact of prescription practices in Makkah on the emergence of antimicrobial resistance. Thus, we recommend further studies in this field that include more cities and centers and with more details on prescription patterns and patient characteristics such as comorbidity and mortality.

## 4. Materials and Methods

### 4.1. Study Design

This retrospective study was conducted to explore antibiotic susceptibility patterns among all *K. pneumoniae* isolates from a tertiary hospital for a duration of eleven years. Samples from January 2011 to December 2021 were included in the study. During that period, the total number of isolates was 61,027. Samples were collected from different wards and included different sample types, such as urine, blood, pus, sputum, swabs, and body fluid. Standard microbiology techniques were performed, and all samples were cultured at 37 °C for 24–48 h in two different media: blood sheep agar and McConkey agar, except for urine samples which were cultured in cystine–lactose–electrolyte-deficient agar (CLED). After 24–48 h, bacterial identification and antibiotic susceptibility patterns were determined using the Vitek-2 (bioMérieux, Marcy-l’Étoile, France) automated system (GN-21341 cards were used for identification, whereas the N291, N292, and N204 cards were used for antibiotic susceptibility) and Microscan walkaway (Beckman Coulter, Brea, CA, USA) automated system (Negative Breakpoint Combo 50, NBC 50). Antibiotics investigated in this study included ampicillin, amoxicillin/clavulanate, piperacillin/tazobactam, ceftazidime, cefotaxime, cefepime, imipenem, gentamicin, amikacin, ciprofloxacin, colistin, tigecycline, and cotrimoxazole. The interpretation of the MIC results relied on Clinical Laboratory Standard Institute guidelines [39]. Identification of ESBL was performed using a simultaneous examination of the inhibitory effects of ceftazidime, cefotaxime, and cefepime, with and without clavulanate.

### 4.2. Statistical Analysis

The total number of patients, specimen type, and antibiograms were entered into a database. Descriptive analysis was conducted to identify the frequency and distribution of all variables. A comparison of antibiotic susceptibility from different years was made using the chi-square test for trends. A *p*-value less than 0.05 was considered statistically significant. Statistical analysis was performed using GraphPad Prism 9.3.0 (GraphPad Software Inc., San Diego, CA, USA).

## Figures and Tables

**Table 1:** The demographic distribution of *K. pneumoniae*.

Year	Total Isolated Organisms	Total *K. pneumoniae* Isolates	Inpatient	Outpatient	Male	Female
2011	8081	622 (7.7%)	546 (87.8%)	76 (12.2%)	348 (55.9%)	274 (44.1%)
2012	7241	677 (9.3%)	528 (78.0%)	149 (22%)	322 (47.6%)	355 (52.4%)
2013	6294	627 (10%)	474 (75.6%)	153 (24.4%)	350 (55.8%)	277 (44.2%)
2014	5217	573 (11%)	471 (82.2%)	102 (17.8%)	323 (56.4%)	250 (43.6%)
2015	5163	727 (14.1%)	581 (79.9%)	146 (20.1%)	436 (60%)	291 (40%)
2016	4722	685 (14.5%)	490 (71.5%)	195 (28.5%)	399 (58.2%)	286 (41.8%)
2017	4376	911 (20.8%)	724 (79.5%)	187 (20.5%)	572 (62.8%)	339 (37.2%)
2018	4980	1131 (22.7%)	994 (87.9%)	137 (12.1%)	679 (60%)	452 (40%)
2019	4835	931 (19.3%)	858 (92.2%)	73 (7.8%)	532 (57.1%)	399 (42.9%)
2020	4029	1043 (25.9%)	893 (85.6%)	150 (14.4%)	681 (65.3%)	362 (34.7%)
2021	6089	1087 (17.9%)	838 (77.1%)	249 (22.9%)	700 (64.4%)	387 (35.6%)

**Table 2:** The proportion of different specimen types from which *K. pneumoniae* was obtained.

		Years										
		2011	2012	2013	2014	2015	2016	2017	2018	2019	2020	2021
Sputum	*K. pneumoniae* isolates	163	173	160	115	135	131	166	236	206	253	224
	Total isolates	1868	1701	1417	1173	1086	912	778	842	785	803	897
	% of *K. pneumoniae*	8.7%	10.2%	11.3%	9.8%	12.4%	14.4%	21.3%	28.0%	26.2%	31.5%	25.0%
Blood	*K. pneumoniae* isolates	83	81	120	176	217	217	335	330	263	409	332
	Total isolates	1107	1094	1075	975	1015	972	1086	1083	1208	1075	2038
	% of *K. pneumoniae*	7.5%	7.4%	11.2%	18.1%	21.4%	22.3%	30.8%	30.5%	21.8%	38.0%	16.3%
Tip and catheter	*K. pneumoniae* isolates	13	12	13	7	15	15	19	15	7	8	11
	Total isolates	149	179	117	78	58	76	67	54	45	40	65
	% of *K. pneumoniae*	8.7%	6.7%	11.1%	9.0%	25.9%	19.7%	28.4%	27.8%	15.6%	20.0%	16.9%
Wound	*K. pneumoniae* isolates	105	144	153	167	166	159	175	237	200	183	228
	Total isolates	1827	1497	1563	1560	1442	1274	1046	1164	1136	881	1183
	% of *K. pneumoniae*	5.7%	9.6%	9.8%	10.7%	11.5%	12.5%	16.7%	20.4%	17.6%	20.8%	19.3%
Urine	*K. pneumoniae* isolates	148	143	97	55	100	104	113	184	136	92	153
	Total isolates	1629	1288	1050	523	674	774	682	918	769	565	948
	% of *K. pneumoniae*	9.1%	11.1%	9.2%	10.5%	14.8%	13.4%	16.6%	20.0%	17.7%	16.3%	16.1%
Miscellaneous	*K. pneumoniae* isolates	110	124	84	53	94	59	103	129	119	98	139
	Total isolates	1696	1482	1064	908	888	714	717	917	892	665	959
	% of *K. pneumoniae*	6.5%	8.4%	7.9%	5.8%	10.6%	8.3%	14.4%	14.1%	13.3%	14.7%	14.5%

**Table 3:** Antibiogram pattern of *K. pneumoniae*.

**Antibiotics**	Year-Wise Prevalence (%) of Resistant *K. pneumoniae*											Total	*p* for Trend
	**2011**	**2012**	**2013**	2014	2015	2016	2017	2018	2019	2020	2021		
Ampicillin	525/558 (94.1%)	592/610 (97%)	454/474 (95.8%)	279/285 (97.9%)	202/206 (98.1%)	282/285 (98.9%)	182/186 (97.8%)	207/210 (98.6%)	231/233 (99.1%)	540/546 (98.9%)	777/782 (99.4%)	4271/4375 (97.6%)	<0.0001
Amoxicillin/ Clavulanate	163/485 (33.6%)	114/284 (40.1%)	272/634 (42.9%)	235/336 (69.9%)	135/274 (49.3%)	249/336 (74.1%)	181/246 (73.6%)	226/297 (76.1%)	170/271 (62.7%)	431/550 (78.4%)	561/775 (72.4%)	2737/4488 (61.0%)	<0.0001
Piperacillin/ Tazobactam	57/419 (13.6%)	290/618 (46.9%)	172/520 (33.1%)	237/334 (71%)	155/201 (77.1%)	208/225 (92.4%)	284/324 (87.7%)	307/320 (95.9%)	214/271 (79%)	422/559 (75.5%)	588/823 (71.4%)	2934/4614 (63.6%)	<0.0001
Ceftazidime	83/278 (29.9%)	216/501 (43.1%)	188/425 (44.2%)	195/288 (67.7%)	236/338 (69.8%)	345/421 (81.9%)	435/534 (81.5%)	623/715 (87.1%)	578/657 (88%)	804/907 (88.6%)	748/881 (84.9%)	4451/5945 (74.9%)	<0.0001
Cefotaxime	64/244 (26.2%)	65/203 (32%)	142/330 (43%)	175/279 (62.7%)	146/247 (59.1%)	422/482 (87.6%)	67/74 (90.5%)	105/124 (84.7%)	136/212 (64.2%)	431/497 (86.7%)	491/577 (85.1%)	2244/3269 (68.6%)	<0.0001
Cefepime	90/167 (53.9%)	86/233 (36.9%)	182/409 (44.5%)	175/230 (76.1%)	241/285 (84.6%)	336/396 (84.8%)	450/523 (86%)	637/712 (89.5%)	584/645 (90.5%)	828/915 (90.5%)	842/981 (85.8%)	4451/5496 (81.0%)	<0.0001
Imipenem	41/623 (6.6%)	73/689 (10.6%)	116/655 (17.7%)	164/428 (38.3%)	243/454 (53.5%)	335/540 (62%)	444/627 (70.8%)	492/856 (57.5%)	390/576 (67.7%)	533/868 (61.4%)	547/913 (59.9%)	3378/7229 (46.7%)	<0.0001
Gentamicin	264/598 (44.1%)	311/689 (45.1%)	310/656 (47.3%)	253/570 (44.4%)	271/699 (38.8%)	411/616 (66.7%)	497/876 (56.7%)	717/1037 (69.1%)	456\900 (50.7%)	672/1028 (65.4%)	614/1037 (59.2%)	4776/8706 (54.9%)	<0.0001
Amikacin	76/641 (11.9%)	94/683 (13.8%)	120/648 (18.5%)	138/440 (31.4%)	158/387 (40.8%)	263/472 (55.7%)	411/726 (56.6%)	645/830 (77.7%)	379/628 (60.4%)	635/938 (67.7%)	605/972 (62.2%)	3524/7365 (47.8%)	<0.0001
Ciprofloxacin	282/609 (46.3%)	338/689 (49.1%)	345/635 (54.3%)	293/413 (70.9%)	270/416 (64.9%)	369/477 (77.4%)	557/678 (82.2%)	755/895 (84.4%)	644/819 (78.6%)	763/918 (83.1%)	739/925 (79.9%)	5355/7474 (71.6%)	<0.0001
Colistin	0/2 (0%)	4/4 (100%)	0/5 (0%)	31/98 (31.6%)	32/184 (17.4%)	40/118 (33.9%)	8/37 (21.6%)	2/2 (100%)	0/4 (0%)	147/501 (29.3%)	105/504 (20.8%)	369/1455 (25.4%)	NS
Tigecycline	0/0 (0%)	31/159 (19.5%)	61/426 (14.3%)	25/179 (14.0%)	15/136 (11.0%)	11/96 (11.5%)	0/49 (0%)	10/10 (100%)	12/97 (12.4%)	90/717 (12.6%)	170/846 (20.1%)	425/2715 (15.7%)	NS
TMP/SMX	240/433 (55.4%)	421/697 (60.4%)	400/648 (61.7%)	312/458 (68.1%)	364/509 (71.5%)	323/455 (71%)	514/656 (78.4%)	763/919 (83%)	689/849 (81.2%)	874/1003 (87.1%)	820/1006 (81.5%)	5720/7633 (74.9%)	<0.0001

The data are presented as the number of resistant isolates/total number of isolates (%). Zero (0) indicates no isolates were tested against the antibiotic. Note: not all isolates were tested against all antibiotics listed here. The *p*-value for trends was calculated using the chi-square test for trends. A *p*-value of less than 0.05 indicates an increase in the resistance trend. NS: Nonsignificant *p*-value (i.e., >0.05).

## Data Availability

Not applicable.
